# Changes in joint coupling and variability during walking following tibialis posterior muscle fatigue

**DOI:** 10.1186/1757-1146-4-6

**Published:** 2011-02-04

**Authors:** Reed Ferber, Michael B Pohl

**Affiliations:** 1 Faculty of Kinesiology, University of Calgary, Calgary, AB, Canada; 2 Faculty of Nursing, University of Calgary, Calgary, AB, Canada

## Abstract

**Background:**

The tibialis posterior muscle is believed to play a key role in controlling foot mechanics during the stance phase of gait. However, an experiment involving localised tibialis posterior muscle fatigue, and analysis of discrete rearfoot and forefoot kinematic variables, indicated that reduced force output of the tibialis posterior muscle did not alter rearfoot and forefoot motion during gait. Thus, to better understand how muscle fatigue affects foot kinematics and injury potential, the purpose of this study was to reanalyze the data and investigate shank, rearfoot and forefoot joint coupling and coupling variability during walking.

**Methods:**

Twenty-nine participants underwent an exercise fatigue protocol aimed at reducing the force output of tibialis posterior. An eight camera motion analysis system was used to evaluate 3 D shank and foot joint coupling and coupling variability during treadmill walking both pre- and post-fatigue.

**Results:**

The fatigue protocol was successful in reducing the maximal isometric force by over 30% and a concomitant increase in coupling motion of the shank in the transverse plane and forefoot in the sagittal and transverse planes relative to frontal plane motion of the rearfoot. In addition, an increase in joint coupling variability was measured between the shank and rearfoot and between the rearfoot and forefoot during the fatigue condition.

**Conclusions:**

The reduced function of the tibialis posterior muscle following fatigue resulted in a disruption in typical shank and foot joint coupling patterns and an increased variability in joint coupling. These results could help explain tibialis posterior injury aetiology.

## Background

Although runners often sustain acute injuries such as ankle sprains and muscle strains, a vast majority of running injuries could be classified as cumulative micro-trauma (overuse) injuries [1-4]. The aetiology of an overuse running injury is multifactorial but muscle fatigue and/or weakness has been discussed as a primary contributing factor [5-9]. Indeed, many lower extremity overuse injuries have been attributed to atypical foot mechanics during gait [10-13]. The tibialis posterior is believed to play a key role in controlling rearfoot eversion [14,15] and providing dynamic support across the midfoot and forefoot during the stance phase of gait [15-17].

The proximal origin of tibialis posterior lies on the interosseous membrane and posterior surfaces of the tibia and fibula. The muscle has multiple distal insertions including the navicular tubercle, the plantar surface of the cuneiforms and cuboid, and bases of the second, third and fourth metatarsals [18]. Biomechanical research conducted on patients with posterior tibialis tendon dysfunction (PTTD) highlights the importance of this muscle in controlling rearfoot, midfoot, and forefoot mechanics during gait [19-21]. However, these studies involved patients with moderate- to advanced-stage PTTD and may not provide adequate information to help us understand the contribution that the tibialis posterior muscle plays in controlling foot pronation in healthy individuals.

One method of assessing a muscle's contribution to a specific movement pattern is via fatigue-inducing exercise of that muscle. Christina et al. [22] showed that localised fatigue of the ankle invertors resulted in a trend towards greater rearfoot eversion during running. However, fatigue of the invertor musculature was achieved using open chain resisted supination exercises, which would result in the recruitment of all invertor muscles including tibialis posterior. Kulig et al. [23] investigated which exercise most selectively and effectively activates tibialis posterior: 1) closed chain resisted foot adduction, 2) unilateral heel raise, and 3) open chain resisted foot supination using MRI to quantify changes in pre- to post-exercise signal intensity. These authors reported that isolated activation of tibialis posterior is best achieved using closed chain resisted foot adduction as opposed to open chain supination. In addition, they reported the greatest tibialis posterior increase occurred with closed chain resisted foot adduction, whereas the mean increase in the other muscles was less than 5%. Therefore, to better understand the role of tibialis posterior fatigue on foot mechanics it seems prudent to use an exercise that more selectively activates and subsequently fatigues this muscle.

Pohl et al. [15] recently conducted a study investigating the effect of localised tibialis posterior muscle fatigue on foot kinematics during walking. These authors reported that following a 30% reduction in tibialis posterior maximal isometric force production, no changes in rearfoot or forefoot kinematics were measured. Specifically, a 0.7 degree increase in peak rearfoot eversion was reported as statistically significant but this change was smaller than the precision error of a within-day gait analysis (0.9 degree). Therefore, these authors postulated the results were not clinically relevant and that it was possible that other muscles, such as tibialis anterior, may have compensated for the lack of tibialis posterior force production thereby resulting in no change in discrete kinematic variables. However, inspection of the data also revealed that 24 out of 29 participants demonstrated an increase in peak rearfoot angle following fatigue (ranging from 0.5 - 2.0 degrees). Since such a consistent change was observed, it raises the question of what other mechanisms and potential explanations can account for these systematic changes. Thus, In light of these findings, it may be worthwhile to investigate the effect of localised muscle fatigue using a joint coupling and coupling variability approach.

The timing or coupling of joint movements has been shown to be a useful tool for understanding injury aetiology based on the notion that asynchrony in joint coupling and changes in joint coupling variability of movement may result in injury [24]. Some researchers have subsequently investigated changes in joint coupling for both injured and healthy participants and reported that, overall, non-injurious coupling involves an in-phase relationship and injurious coupling involves more out-of-phase joint coupling relationship throughout stance [5,25-27]. However, these studies have focused primarily on thigh:shank or rearfoot:shank couplings in an effort to understand knee-related injuries. Moreover, these studies have utilised a cross-sectional approach and compared the joint coupling and/or coupling variability patterns between injured and non-injured groups. Few studies have investigated the complexities of the multiple foot segments using a joint coupling approach or by investigating joint coupling variability.

Variability in joint coupling has been suggested to play a role in the aetiology of injury. Hamill et al. [24] proposed that injured runners exhibit reduced joint coupling variability thereby reducing the flexibility in the system and increasing the potential for musculoskeletal injury. Other studies have supported this finding for patients with iliotibial band syndrome [28] and for female runners as a possible mechanism to explain the higher incidence of ACL injuries compared to males [29]. Moreover, Miller et al. [28] suggested that muscle dysfunction and/or weakness may be a possible explanation for the reduced joint coupling variability measured after an exhaustive run for a group of injured runners. However, these authors did not measure changes in muscle strength following the run and the aforementioned studies [28,29] utilised a cross-sectional approach and/or extrinsic perturbations to investigate changes in joint coupling variability. To our knowledge, no study has utilised a muscle fatigue protocol (an intrinsic perturbation) to better understand potential changes in joint coupling and/or joint coupling variability to shed light on injury aetiology.

Therefore, the purpose of this study was to examine the effect of localised tibialis posterior muscle fatigue on shank, rearfoot and forefoot joint coupling and coupling variability during walking. It was hypothesised that following a bout of fatigue-inducing exercise participants would demonstrate altered and non-synchronous joint coupling between the respective segments. Since no study has specifically investigated changes in joint coupling for the ankle and foot segments in such a manner, we chose to leave this hypothesis non-directional. Since several other muscles, specifically tibialis anterior, flexor hallucis longus, flexor digitorum longus, and peroneus longus, also serve to control foot and ankle kinematics, it is reasonable to assume that localised fatigue of one muscle would force the supporting musculature to increase their role in maintaining a normal mechanical pattern. Since fewer muscles are now functioning to perform a given task, we hypothesised that following fatigue a reduction in coupling variability would be measured. We also hypothesised that the greatest changes in joint coupling and variability would occur at or near midstance when loading to the foot and shank would be greatest.

## Methods

### Participants

Twenty-nine (11 males, 18 females) recreationally active participants (age = 27.3 ± 8.1 years; mass = 68.8 ± 13.5 kg; height = 172.8 ± 13.5 cm) volunteered to participate in the study. All participants were currently free from lower extremity injury, had no prior history of surgery, and were familiar with treadmill walking. The study was approved by the institutional ethics board, and written informed consent was obtained from all participants.

### Procedures

More in depth explanations of the procedures and methods can be found in a previous publication [15]. In brief, three-dimensional kinematic data were collected for all participants walking barefoot on a treadmill both prior to, and following, fatigue-inducing exercise of the tibialis posterior muscle of the right limb. Seventeen reflective markers (9 mm diameter) were attached to the skin of the forefoot, rearfoot and shank as described previously [15,30]. Kinematic walking data were collected at 120 Hz using an eight-camera motion analysis system (Vicon Motion Systems Ltd, Oxford, UK) arranged around a treadmill (StarTrac, Irvine, USA). A standing static calibration trial was recorded followed by walking on a treadmill at 1.1 ms^-1^. Subjects were provided 2-3 minutes to accommodate to the treadmill and the speed chosen. Once accommodated and comfortable on the treadmill, ten footfalls of kinematic data were collected to represent the "pre-fatigue" (PRE) condition. Upon completion of the fatigue exercise protocol, participants immediately completed the "post-fatigue" (POST) walk and another 10 footfalls were collected.

Muscle fatigue was defined as a reduction in the capacity of the muscle to perform work or generate force [15,22]. Participants were seated in a chair while their right foot was placed in a custom built device containing a dynamometer (Lafayette Instrument, Lafayette, USA: Model 01163) that 1) allowed participants to perform concentric/eccentric foot adduction contractions with adjustable resistance and 2) enabled the measurement of a maximal voluntary isometric contraction (MVIC) during foot adduction. The mean of three MVIC trials was taken to represent baseline strength. Then, participants performed sets of 50 concentric/eccentric contractions at 50% MVIC through a 30° range of motion with 10 seconds of rest between each set. MVICs were repeated after every four sets and exercises were continued until participants MVICs had dropped below 70% of the pre-fatigue values or they were unable to complete two consecutive sets. A final set of MVICs were taken immediately following the post-fatigue walk (within 2 minutes) to determine whether participants had recovered in strength during the walking trial.

### Data processing

Ten foot falls for the PRE and POST kinematic walking data were selected for analysis. Raw kinematic data were filtered using a fourth order low-pass Butterworth filter at 12 Hz. Anatomical co-ordinate systems for the shank, rearfoot and forefoot, along with three-dimensional segment angles were calculated using Visual 3 D software (C-motion Inc, Rockville, USA) [15,31]. All segment angles were defined as motion measured relative to the next most proximal segment [19,21] and the segment angles during walking were expressed relative to the standing calibration trial. All kinematic data were analysed for the stance phase and normalised to 101 data points. Initial contact (IC) and toe off (TO) were identified using a kinematic velocity-based algorithm [32] applied to the posterior calcaneal and dorsal phalanx markers respectively. Custom Labview (National Instruments Corp, Austin, USA) software was used to extract the kinematic coupling variables of interest for each subject. Specifically, the following joint coupling and coupling variability relationships were investigated: 1) tibia internal/external rotation:rearfoot inversion/eversion (TIBrot:RFi/ev), 2) rearfoot inversion/eversion:forefoot dorsi/plantarflexion (RFi/ev:FFd/pf), and 3) rearfoot inversion/eversion:forefoot abd/adduction(RFi/ev:FFab/d). We chose these joint coupling relationships to compare the results with previous studies [5,26,28,30,31].

Angle-angle plots of proximal and distal segments for each trial were created. The coupling angle was determined using a modification of a vector coding technique suggested by Heiderscheit et al. [33]. The absolute resultant vector between two adjacent data points during the stance phase of running was calculated (equation 1) and, following conversion from radians to degrees, the resulting range of values for coupling angle was 0-90°.

(1) $${Ø}_{\text{i}}=\text{abs}[{\text{tan}}^{-\text{1}}({\text{y}}_{i+\text{1}}–{\text{y}}_{i}/{\text{x}}_{i+\text{1}}–{\text{x}}_{i})]$$

where *i *= 1,2, and n

Thus, with the distal segment motion plotted on the abscissa and proximal segment motion plotted on the ordinate, a coupling angle of 45° would indicate equal amounts of segmental motion. An angle greater than 45° indicates greater proximal segment motion relative to distal segment. Similar to previous studies [24,26], for the purpose of analyzing the coupling angles and variability within specific regions of stance, each relative motion plot was first normalized to 100% of stance and then divided into 4 phases. Phase 1 ranged from heel strike to 25% of stance, phase 2 from 25-50% of stance, phase 3 from 50-75% of stance, and phase 4 from 75-100% of stance. To calculate the average coupling angle values for each phase of stance, each data point was averaged on a point-by-point basis across the ten trials resulting in an average trace. From the average trace, the average coupling angle for each phase of stance was calculated over time. The standard deviation was calculated on a point-by-point basis across the 10 trials and the between-trial, within-subject joint coupling variability for each phase of stance was calculated across time for each phase of stance.

### Data analysis

Group descriptive statistics were calculated for each variable for both PRE and POST fatigue conditions. Paired sample t-tests (two-tailed) were conducted for the variables of interest for between-condition statistical comparison. Since we hypothesised that the greatest changes in coupling and variability would occur at or near midstance, a priori t-tests were performed on phase 2 and phase 3 data and significance for these tests was set at an alpha level of *P *< 0.05. If necessary, analysis of phases 1 or 4 were performed to help better understand our results and an alpha level of *P *< 0.01 was established to minimize type I error. All analyses were undertaken using SPSS 15.0 (SPSS Inc, Chicago, USA).

## Results

### Strength

As reported previously, following the fatigue exercise protocol the MVIC strength dropped to 67% of the baseline values (p = 0.001; PRE = 66.2 N; POST = 44.6). Eight participants did not drop below the predetermined threshold of 70% baseline MVIC but were still included in the analysis since they were unable to complete two additional sets of 50 repetitions due to muscle fatigue and also exhibited a 21% reduction in force output. Immediately following the post-fatigue walk, the MVIC strength was 80% of the baseline.

### Joint Coupling

**Figure 1:**
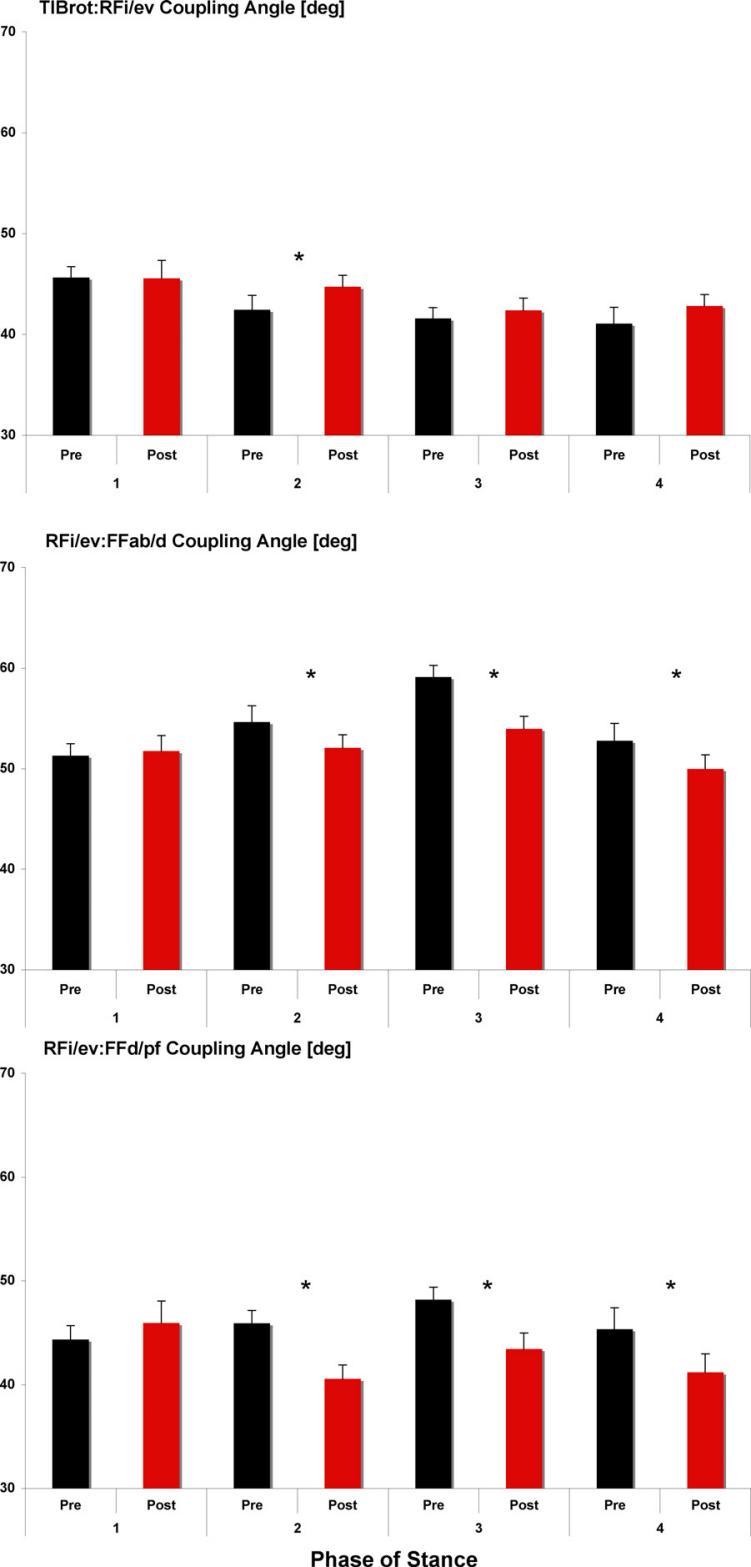
**Joint coupling angle prior to and following the fatigue protocol and across phase of stance**. Note, * indicates *P *< 0.05.

A summary of pre- and post-fatigue changes in TIBrot:RFi/ev, RFi/ev:FFd/pf, and RFi/ev:FFab/d joint coupling angle is provided in Figure 1 and Table 1. For TIBrot:RFi/ev, a significant increase in joint coupling angle during Phase 2 (p = 0.05; PRE = 42.42°; POST = 44.71°) was measured following the fatigue protocol. RFi/ev:FFd/pf significantly decreased during Phase 2 (p = 0.04; PRE = 45.91°; POST = 40.58°) and Phase 3 (p = 0.01; PRE = 48.19°; POST = 43.42°) and RFi/ev:FFab/d, also significantly decreased during Phase 2 (p = 0.01; PRE = 54.62°; POST = 52.06°) and Phase 3 (p = 0.01; PRE = 59.09°; POST = 53.94°) compared to pre-fatigue values.

**Table 1:** Summary of shank, rearfoot, and forefoot joint coupling and coupling variability (Mean, (SD)) prior to (PRE) and following fatigue (POST).

Coupling Angle (deg)	Phase 1		Phase 2		Phase 3		Phase 4	
	
	PRE	POST	PRE	POST	PRE	POST	PRE	POST
TIBrot:RFi/ev	45.63	45.54	42.42	44.71*	41.59	42.36	41.06	42.82
	(5.74)	(5.68)	(9.58)	(6.66)	7.78	8.73	6.11	6.11
RFi/ev:FFd/pf	44.34	45.92	45.91	40.58*	48.19	43.42*	45.32	41.17*
	(7.12)	(6.47)	(11.50)	(8.35)	6.63	11.14	7.02	9.55
RFi/ev:FFab/d	51.30	51.72	54.62	52.06*	59.09	53.94*	52.76	49.97
	(6.22)	(6.38)	(8.47)	(6.72)	8.68	9.24	7.05	7.48
								

**Coupling Variability (deg)**	**Phase 1**		**Phase 2**		**Phase 3**		**Phase 4**	
	
	**PRE**	**POST**	**PRE**	**POST**	**PRE**	**POST**	**PRE**	**POST**

TIBrot:RFi/ev	23.81	23.41	20.66	22.34*	18.71	20.84*	16.88	20.11*
	(2.78)	(1.55)	(3.10)	(2.99)	3.89	4.15	3.15	3.35
RFi/ev:FFd/pf	25.11	24.57	23.04	23.58	22.63	22.88	22.24	22.62
	(1.79)	(2.15)	(3.22)	(2.57)	4.48	3.42	4.16	2.40
RFi/ev:FFab/d	24.15	24.16	21.34	23.37*	20.64	22.94*	19.89	21.16
	(2.32)	(1.96)	(4.01)	(2.50)	4.93	3.84	4.38	2.48

* indicates p < 0.05

### Coupling Variability

**Figure 2:**
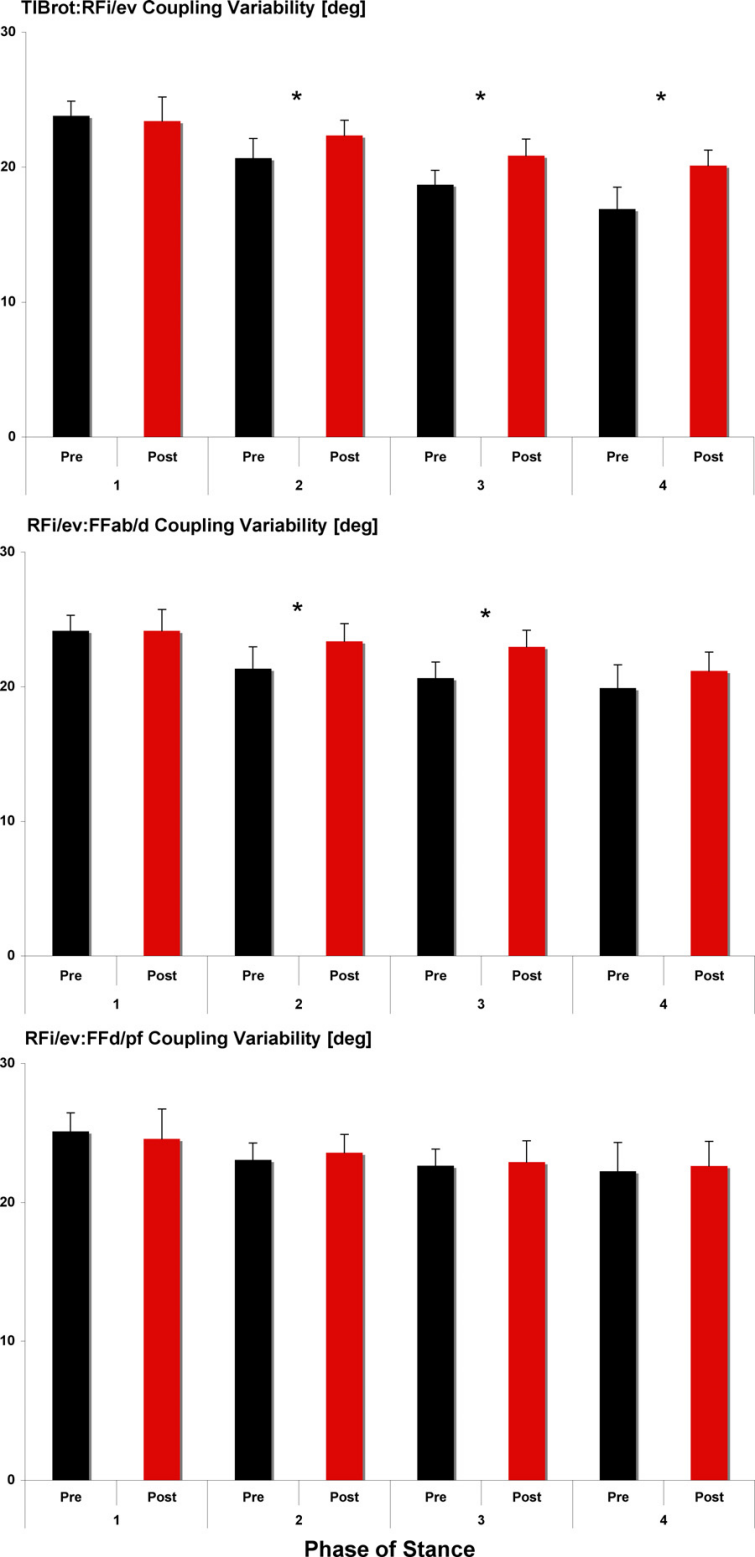
**Joint coupling variability prior to and following the fatigue protocol and across phase of stance**. Note, * indicates *P *< 0.05.

A summary of pre- post-fatigue changes in TIBrot:RFi/ev, RFi/ev:FFd/pf, and RFi/ev:FFab/d joint coupling variability is provided in Figure 2 and Table 1. TIBrot:RFi/ev significantly increased during Phase 2 (p = 0.01; PRE = 20.66°; POST = 22.34°) and Phase 3 (p = 0.01; PRE = 18.71°; POST = 20.84°) following the fatigue protocol. A significant increase in RFi/ev:FFab/d joint coupling variability was measured for Phase 2 (p = 0.01; PRE = 21.34°; POST = 23.37°) and Phase 3(p = 0.01; PRE = 20.64°; POST = 22.94°) compared to pre-fatigue values. No changes in RFi/ev:FFd/pf variability were measured across any phase compared to pre-fatigue values (Table 1).

## Discussion

The purpose of this study was to examine the effect of localised tibialis posterior muscle fatigue on shank, rearfoot, and forefoot joint coupling and coupling variability during walking. Two main hypotheses were put forth: following a bout of fatigue-inducing exercise participants would demonstrate 1) altered and non-synchronous joint coupling between the respective segments as well as 2) reduced coupling variability. To test these hypotheses, a unique approach was utilised to selectively fatigue the tibialis posterior muscle.

The protocol for reducing the force output of this muscle was developed based on Kulig et al. [24] who showed that isolated activation of tibialis posterior is best achieved using closed chain resisted foot adduction. The results of the present study indicate that this fatigue protocol was successful in reducing the MVIC force by over 30%. Although eight participants did not achieve the targeted 30% reduction in force production, they did all achieve at least a 21% reduction and were unable to complete 2 consecutive sets of the 50-repetition exercise. Furthermore, there was no evidence that these eight participants differed systematically from the rest of the sample in terms of kinematic changes following fatigue based on the results of the current study and previous study [15]. Finally, the reduction in strength was still apparent following the POST data collection indicating that the fatigue protocol was effective.

The decrements in isometric force are similar to previous fatigue studies and studies involving healthy runners and PTTD patients. Cheung and Ng [34] reported similar findings for fatigue of the invertor muscles in healthy runners following an exhaustive run. Moreover, Alvarez et al. [35] reported a 40% reduction in concentric ankle invertor strength for PTTD patients prior to a 16-week rehabilitation program. However, it should be recognized that the PTTD patients in this study included advance-stage PTTD patients who had symptoms for approximately 16.5 weeks prior to treatment. Pilot data from our laboratory shows that early-stage PTTD patients exhibit a 17% reduction in ankle invertor MVIC strength compared to healthy controls. Thus, we are confident that our fatigue protocol and a priori criteria for localised muscle fatigue is sufficient to induce tibialis posterior muscle fatigue and concomitant reductions in force output during a dynamic task such as walking.

In support of the first hypothesis, a change in joint coupling angle between 2.3° and 5.3° was measured during Phase 2 and 3. Moreover, the tibia and forefoot all increased their respective motions relative to the rearfoot. While we are not aware of another study that has investigated changes in foot and ankle joint coupling following a fatigue protocol, the pre- and post-fatigue coupling angle data in the current study are similar to Pohl et al. [31] who also reported joint coupling angles at or near 45° for the same coupling relationships while walking. Specifically, the pre-fatigue values for the TIBrot:RFi/ev coupling angle indicate a near 1:1 ratio in coupling for Phase 1 then greater overall motion of the rearfoot throughout the remainder of stance which is similar to previous studies [25,26].

Post-fatigue, and during Phase 2 of stance, increased tibial motion was measured, relative to the rearfoot, suggesting that fatigue of the tibialis posterior disrupted the typical coupling mechanics between these two segments. This same relationship was observed for the RFi/ev:FFd/pf coupling relationship wherein a 1:1 coupling relationship is measured pre-fatigue and post-fatigue shows a change in forefoot motion relative to the rearfoot for Phases 2 and 3. Finally, greater overall rearfoot motion, relative of the forefoot (RFi/ev:FFab/d), was measured pre-fatigue, which is consistent with previous studies [31], and fatigue of the tibialis posterior disrupted this relationship resulting in greater motion of the forefoot relative to the rearfoot for Phases 2 and 3 of stance. Thus, it can be concluded that when the tibialis posterior is unable to produce sufficient force, there are significant alterations in coupling patterns for the shank and foot.

We postulate that the overall greater motion of the tibia and forefoot (relative to the rearfoot) following fatigue is the result of the functional anatomy of the tibialis posterior muscle itself. The tibialis posterior muscle originates from the tibia and the tendon does not attach directly to the rearfoot (calcaneus), but has several attachment points to the midfoot and forefoot. Thus, it is reasonable to speculate that greater relative motion of these segments is the result of the inability of the muscle, via fatigue and reduced force output, to control the individual motions of these foot segments.

In contrast to the second hypothesis, an increase in joint coupling variability was measured following the fatigue protocol for TIBrot:RFi/ev and RFi/ev:FFab/d during Phase 2 and 3. These results are in contrast to several other studies investigating joint coupling variability. Ferber et al. [26] studied different types of orthotics during running and reported no significant changes in TIBrot:RFi/ev variability across orthotic conditions or compared to a control group. Hamill et al. [24] studied patients with patellofemoral pain syndrome (PFPS) and reported overall reduced joint coupling variability for thigh and shank coupling variability compared to the uninjured leg and a control group. However, these authors measured thigh and shank coupling patterns or variability so it is difficult to compare their results with those of the present study. Also in contrast to the results of the present study, Miller et al. [36] reported that runners with a history of iliotibial band syndrome (ITBS) demonstrated reduced TIBrot:RFi/ev coupling variability while running on a treadmill compared to a control group. However, it is important to note that these studies involved runners who were either injured at the time of testing or had a long history of running-related injuries. The participants in the current study were healthy athletes with no history of chronic injury and involved an intrinsic perturbation rather than a cross-sectional comparison. In addition, these authors [24,36] used a different measure for coordination (continuous relative phase), which may not directly compare to the present vector coding method and could explain the different findings of the present study. Thus, comparisons to previous studies must be made with caution.

While we are not aware of another study utilising a muscle-fatigue protocol to measure changes in either joint coupling or coupling variability, two studies have investigated changes in movement variability following an intervention of some type. Ferber et al. [37] reported that following a 3-week strengthening protocol, a reduced variability in stride-to-stride knee joint kinematic patterns was adopted by the PFPS group. These authors suggested that, from a clinical perspective, restoration of a more consistent and predictable movement pattern is expected with the increases in muscle strength and reductions in pain. Thus, the increase in coupling variability following fatigue in the present study is consistent with these authors [37]. Further research involving changes in coupling variability following successful rehabilitation from a musculoskeletal injury is, however, warranted.

Few studies have investigated the effect of fatigue on changes in joint coupling. Miller et al. [28] studied changes in joint coupling variability during an exhaustive run for runners who had previously experienced ITBS. Compared with the control group, the ITBS runners were more variable in knee flex/extension-foot add/abduction at the start of the run, less variable in thigh add/abduction-foot inv/eversion at the end of the run, tended to be less variable in thigh add/abduction-tibia rotation at the end of the run but showed no change in TIBrot:RFi/ev coupling variability either during the entire stride cycle, swing, or stance phase. It is possible that since a variety of changes in coupling variability were reported, albeit the majority of joint coupling relationships showing reduced variability, that increased coupling variability for the shank and foot is a possible mechanism to explain injury aetiology similar to the results of the present study.

Based on the redundancy of the various muscles that serve to control frontal plane rearfoot and transverse plane tibial motion, a potential strategy for the foot may be to increase coupling variability to avoid injury. We postulate that a diminished ability of the muscle to produce a vigorous contraction, a concomitant reduction in joint contact force, and a resulting increase in joint coupling variability could result. In other words, the reduced function of the tibialis posterior muscle following fatigue would result in less control of joint movement since fewer muscles are functioning to achieve a desired movement pattern. Moreover, since tibialis posterior is a major invertor of the foot, and we successfully fatigued this muscle, other muscles must compensate to control foot pronation. Given these muscles are not as accustomed to localised fatigue conditions, this might also contribute to the increased variability that was observed. Finally, it is possible that reduced posterior tibialis function lead to increased activation levels of other inverters with the goal of compensating for the loss of force. Future studies are needed to improve our understanding of the lower extremity as a dynamical system in healthy and injured runners and how kinematic coupling and variability patterns may change for patients with chronic and more advanced PTTD.

There are factors that may have influenced the results of this study. While closed-chain foot adduction has been shown to be the best exercise at selectively activating tibialis posterior [23], as previously discussed, other muscles also play a role in this movement. Therefore, this study was limited in its ability to specifically quantify the degree of fatigue that was achieved in the tibialis posterior muscle. An alternative approach would be to quantify changes in muscle activity and fatigue via the use of electromyography [38]. Subsequent EMG studies would also enable greater understanding of the compensation strategies employed by other muscles. Second, the order of conditions was the same for all subjects and ideally the order would be balanced to minimize the changes of a presentation bias. However, in a fatigue study, it is admittedly difficult to achieve randomization of order unless EMG, MRI, or some other valid measurement technique was used to ensure that participants recovered from the fatigue protocol prior to post-fatigue testing. Third, we chose to investigate potential changes in joint coupling and coupling variability using a vector coding technique. However, other techniques, such as continuous relative phase (CRP) are also available. Perhaps using a method such as CRP would yield different results but Miller et al. [39] stated that both vector coding and CRP methods seem to be valid metrics for assessing variability. However, future research using different methods of assessing joint coupling is warranted. Finally, our analysis was restricted to the stance phase of gait and did not include the swing phase. Previous studies have reported differences in coordination variability during swing or during the transitions between swing to stance [24,28,33]. However, since the ankle invertor muscles exhibit minimal or no activity during the swing phase of gait [16], we chose not to analyze these data.

## Conclusions

Following a repeated bout of exercise, a fatigue protocol was successful in reducing the MVIC force of the tibialis posterior muscle by over 30%. Concomitant with the reduction in force output was a change in joint coupling patterns and increase in coupling variability. We conclude that once the tibialis posterior muscle was fatigued, fewer muscles are functioning to achieve a desired movement pattern and alterations in joint coupling and coupling variability result. These changes could help explain tibialis posterior injury aetiology and serve to optimize injury rehabilitation.

## Competing interests

The authors declare that they have no competing interests.

## Authors' contributions

MBP and RF developed the rationale for the study, designed the study protocol, conducted the data collections, processed the data, and drafted the manuscript. All authors have read and approved the final manuscript.
